# Audiological Phenotypes of Connexin Gene Mutation Patterns: A Glance at Different GJB2/GJB6 Gene Mutation Profiles

**DOI:** 10.3390/children11020194

**Published:** 2024-02-03

**Authors:** Leonardo Franz, Alessandro Incognito, Chiara Gallo, Licia Turolla, Elisa Scquizzato, Roberta Cenedese, Alessandro Matarazzo, Daniel Savegnago, Paolo Zanatta, Elisabetta Genovese, Cosimo de Filippis, Gino Marioni

**Affiliations:** 1 Phoniatrics and Audiology Unit, Department of Neuroscience DNS, University of Padova, 35122 Treviso, Italy; leonardo.franz@unipd.it (L.F.); alessandro.incognito@aulss2.veneto.it (A.I.); chiara.gallo@aulss2.veneto.it (C.G.); roberta.cenedese1@aulss2.veneto.it (R.C.); daniel.savegnago@studenti.unipd.it (D.S.); cosimo.defilippis@unipd.it (C.d.F.); 2 Medical Genetics Unit, Treviso Hospital, 31100 Treviso, Italy; elichia248@gmail.com; 3 Molecular Pathology Laboratory, Unit of Pathological Anatomy, Treviso Hospital, 31100 Treviso, Italy; elisa.scquizzato@aulss2.veneto.it; 4 Department of Anesthesiology and Critical Care, Treviso Hospital, 31100 Treviso, Italy; paolo.zanatta1@aulss2.veneto.it; 5 Otorhinolaryngology Unit, Department of Medical and Surgical Sciences for Children and Adults, University of Modena and Reggio Emilia, 41121 Modena, Italy; elisabetta.genovese@unimore.it

**Keywords:** genetic hearing loss, connexin, GJB2/GJB6 gene mutation, audiological phenotype

## Abstract

GJB2 mutations are the most common cause of autosomal-recessive non-syndromic sensorineural hearing loss (SNHL). The available evidence shows large phenotypic variability across different genotypes and allelic variants. The aim of this study was to investigate the clinical and audiological features of a cohort of subjects with different GJB2/GJB6 gene mutation profiles from a tertiary referral center in Northeastern Italy. We considered 57 patients with GJB2/GJB6 mutations presenting with congenital, non-syndromic SNHL, mainly coming from the Veneto region (Italy). The samples were screened for mutations in exons 1 and 2 of the GJB2 gene and for the GJB6 gene deletion del (GJB6-D13S1830). Free-field and air-conduction frequency-specific thresholds and the pure-tone average (PTA) were considered in the statistical analysis. Five patients (8.87%) had connexin gene mutations in simple heterozygosis, 15 (26.31%) in compound heterozygosis, 34 (59.64%) in homozygosis, and 3 (5.26%) with digenic patterns. The frequency-specific air-conduction thresholds showed significantly different mean values across the different genotypes (Roy’s largest-root test, *p* = 0.0473). Despite the evidence already available on genetic SNHL, many new insights are to be expected. Further large-scale prospective studies including different populations are necessary to confirm these preliminary findings about the clinical and audiological features of patients with different GJB2/GJB6 gene mutation patterns.

## 1. Introduction

Congenital hearing impairment (HI) has been reported to be the most common birth defect, with an incidence of 1–3 out of every 1000 infants [1,2]. HI can have multifactorial etiology linked to genetic factors or environmental causes. Almost 50% of HI is due to genetic factors [3] that may be syndromic or non-syndromic. The syndromic form is associated with anomalies of other body parts and pathological manifestations affecting other organs or systems, and accounts for about 30% of hereditary HI [4]. The non-syndromic form (70%) is not associated with any other defect in the body and its functions, only concerning HI, and may have autosomal-recessive, autosomal-dominant, X-linked, or mitochondrial inheritance, with high genetic and allelic heterogeneity [5].

Mutations in GJB2, the gene encoding the gap junction protein connexin 26 (Cx26), are the most frequent cause of autosomal-recessive, non-syndromic, sensorineural hearing loss (SNHL) in many populations across the world [6]. Connexin’s functions are crucial for intercellular communication. In particular, Cx26 regulates the K^+^ ion recirculation processes, allowing electrical and metabolic cellular coupling, which generates the endo-cochlear potential [7]. Cx26 and connexin 30 (Cx30), the latter encoded by the GJB6 gene, are co-expressed in many tissues, including the cochlea, and they are capable of forming functional heteromeric channels [8]. GJB2 gene mutations account for up to 50% of non-syndromic deafness worldwide [9]. Several allelic mutations have been associated with SNHL, with different prevalence in different geographical areas and in specific ethnic groups. Pathogenic variants include nonsense, small insertion or deletion, missense, and splicing variants, but for many of these variants, the mechanism by which they cause the phenotypes still need to be fully characterized [10,11]. In Europe, one of the most common pathogenic variants is 35delG of the GJB2 gene [12], with a carrier frequency in the European population of between 1 and 4% [13]. A large deletion involving most of the GJB6 gene is the second most common connexin mutation in many populations [14,15,16].

Since sequencing of the coding region of the GJB2 and GJB6 genes has become a routine investigation in many centers, it has been possible to observe the clinical phenotypic variability associated with these mutations [5,17,18,19,20,21,22]. Indeed, in subjects with different pathogenic connexin mutations, the degree of hearing loss can vary from mild to profound, the time of onset can be congenital or arise in late childhood, and the trend can be stable or progressive [17,23].

The main aim of this investigation was to analyze the clinical and audiological features of a cohort of subjects with different GJB2/GJB6 gene mutation profiles. The preliminary hypothesis is that phenotypically different audiological scenarios can be associated with different genotypes.

## 2. Materials and Methods

### 2.1. Patients

This study was conducted according to principles of the Helsinki Declaration. All enrolled patients or their parents signed an informed consent form regarding the anonymized processing and publication of their data. Data were evaluated in agreement with Italian privacy and sensitive data laws and the regulations of Padova University.

During the period from November 2014 to July 2023, 335 patients with suspected non-syndromic genetic SNHL were investigated by our group. Among these, we analyzed a series of 57 patients with congenital, non-syndromic, GJB2/GJB6 genetic mild-to-profound SNHL, primarily from the Veneto region (Italy), but also from other regions and countries, amounting to 114 hearing-impaired ears.

For the statistical analysis, patients were divided into four groups based on their genotypes as follows:

(i) Simple heterozygosis: subjects with a mutation in a single allele of the GJB2 gene.
(ii) Compound heterozygosis: patients who had two different mutations in the respective alleles of the same gene (in the case of this study, GJB2).
(iii) Homozygosis: patients who had the same mutation in both alleles of the same gene (in the case of this study, GJB2).
(iv) Digenic: patient who had a mutation in both GJB2 and GJB6.

Table 1 summarizes the main demographic and statistical data, stratified by genotype groups, according to gender, age at diagnosis, familiarity, natural history (progressive or stable), and PTA based on the test examination results. All patients had either hearing aids, a unilateral cochlear implant with a contralateral hearing aid, or only a cochlear implant.

Patients who met the following criteria were considered eligible for this study: (i) children or adults born in Italy or abroad with a primary diagnosis of congenital, non-syndromic, GJB2/GJB6 genetic, mild-to-profound SNHL made at the Phoniatrics and Audiology Unit of Padova University, Treviso, Italy; (ii) homozygous, compound heterozygous, and double heterozygous individuals with monoallelic mutation of both GJB2 and GJB6 genes. Exclusion criteria were as follows: (i) patients with simple heterozygosis for deletion in the gene encoding for Cx30; (ii) patients with syndromic hearing loss; (iii) patients with acquired hearing loss as a result of known pre-, peri-, or post-natal ototoxic insult due to viral infection, jaundice, meningitis, drugs, or other conditions; (iv) and patients with genetic mutations including variants of unknown meaning.

### 2.2. Audiological Investigations

According to the Year 2019 Position Statement, the principles and guidelines for Early Hearing Detection and Intervention Programs Audiological investigations [24] in children were based on the following:

(i) From birth to 3 months of age: Auditory Brainstem Response (ABR), Transient Evoked Otoacoustic Emissions (TEOAE), Distortion Product OAEs (DPOAE), and Tympanometry and Acoustic Reflex (impedancemetry).
(ii) From 3 to 8 months of age: BOA (Behavioral Observation Audiometry) and ABR/OAEs according to collaboration.
(iii) From 8 to 24 months of age: Visual Reinforcement Audiometry (VRA).
(iv) From 2 to 3 years of age: Play Audiometry and Peep Show.

We applied hearing aids to all children from 3 months or cochlear implants from 12 to 18 months, and the audiological investigations were performed with and without hearing aids/cochlear implants.

VRA and play audiometry were performed according to the procedures recommended by the British Society of Audiology [25].

Starting from 5 years of age, a diagnostic test was performed with a tonal audiometric test with the use of headphones in a sound booth, with and without hearing aids/cochlear implants, tympanometry, and Acoustic Reflex (impedancemetry) at all ages.

Speech audiometry was based on an Italian adaptation of the English speech perception test [26].

We also used impedance test to exclude middle-ear diseases and, whenever possible, to detect the stapedial reflex threshold.

### 2.3. Genetic Investigations

DNA was extracted from buccal swab samples using a QIAamp DNA QIAcube Kit (QIAGEN, Hilden, Germany). Samples were screened for mutations in exons 1 and 2 of the GJB2 gene and for the GJB6 gene deletion del (GJB6-D13S1830). Exon 1 and exon 2 sequencing was performed using the ABI BigDye Terminator v1.1 Cycle Sequencing Kit (Applied Biosystems, Inc., Foster City, CA, USA) on an ABI 3500 Sanger sequencing platform. The list of primers and conditions used for PCR and sequencing reactions is available upon request. DNA sequence variations were found by comparing the subject DNA sequence to the GJB2 reference sequence NG_008358.1. Electropherograms were analyzed by visual inspection and pairwise alignment to reference sequences through SeqScape software version 2.7 (Applied Biosystems, Inc., Foster City, CA, USA) and six different primers (Invitrogen by Thermo Fisher Scientific Inc., Foster City, CA, USA) according to del Castillo et al. [27].

The test for the detection of del (GJB6-D13S1830) was adapted according to del Castillo et al. [27]. PCR was conducted using AmpliTaq Gold 360 Master Mix (Applied Biosystems, Inc., Foster City, CA, USA). Separation of the PCR products was performed using electrophoresis in a 3% agarose gel.

The above-described tests led to the identification of four different genotypes: simple heterozygosis, compound heterozygosis, homozygosis, and digenic mutations.

The clinical interpretation of variants other than 35delG of the GJB2 gene was performed according to the databases of variants of the GJB2 gene [28,29] and to the ClinVar and Varsome databases [30,31] or according to the ACMG criteria for the interpretation of sequence variants [32].

### 2.4. Statistical Analysis

Multivariate analyses of the means and variances of hearing thresholds at 250, 500, 1000, 2000, and 4000 Hz across different genotypes were performed according to a MANOVA model using Roy’s largest-root test. Upon applying a MANOVA model based on 4 groups to the 114 hearing-impaired ears, with an estimated between-group minimum variance of 0.1, the minimum statistical power was calculated to be 0.80.

The MANOVA model, with chi-squared approximation, was also used to investigate the distribution of other continuous variables across different genotypes.

Fisher’s exact test was used for categorical variables.

The characteristics of hearing threshold distribution were explored using a skewness univariate normality test.

A *p*-value of <0.05 was considered significant. The statistical package STATA 16.1 (Stata Corp LP, College Station, TX, USA) was used for all analyses.

## 3. Results

### 3.1. General Clinical Outcomes

A total of 57 patients, 26 males and 31 females (mean age at diagnosis: 5.4 ± 3.5 years), amounting to 114 hearing-impaired ears, were included in this investigation.

The skewness univariate normality test could not lead us to reject the null hypothesis of a normal distribution of hearing thresholds for all frequencies (*p* > 0.05), except for 2000 Hz, which seemed to show a non-normal distribution (skewness univariate normality test, *p* = 0.035).

Overall, the average free-field PTA was 58.6 ± 14.8 dB. Considering both ears for each patient included, the average air-conduction PTA was 62.0 ± 29.3 dB. Considering cases with available data on bone conduction, the mean air–bone gap was 4.8 ± 0.9 dB.

After a mean follow-up time of 72.89 ± 160.37 months, the mean difference between air-conduction PTA at the last clinical evaluation and at the first available hearing test was 0.01 ± 9.66 dB. Overall, only in nine affected ears (15.79%) was progressive hearing loss noticed.

**Figure 1:**
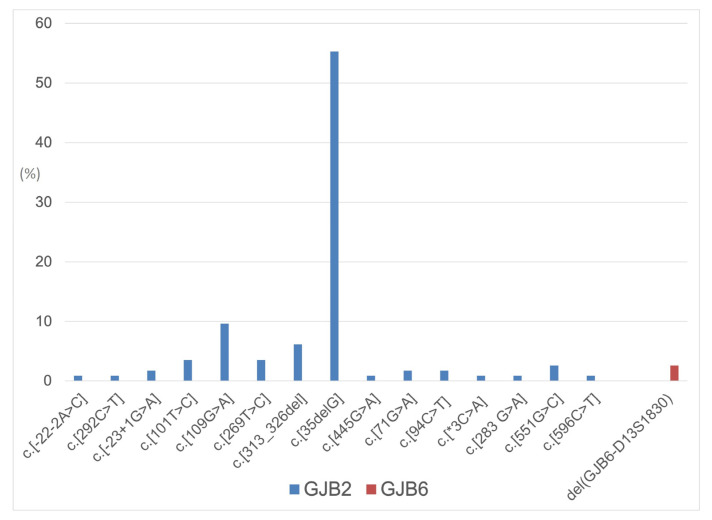
Distribution of GJB2 and GJB6 allelic variants in the considered population.

Regarding genotype classification, 5 patients (8.87%) had connexin gene mutations in simple heterozygosis, 15 (26.31%) in compound heterozygosis, 34 (59.64%) in homozygosis, and 3 (5.26%) with a digenic pattern (see also Figure 1).

### 3.2. Association between Genotype and Clinical Features

**Figure 2:**
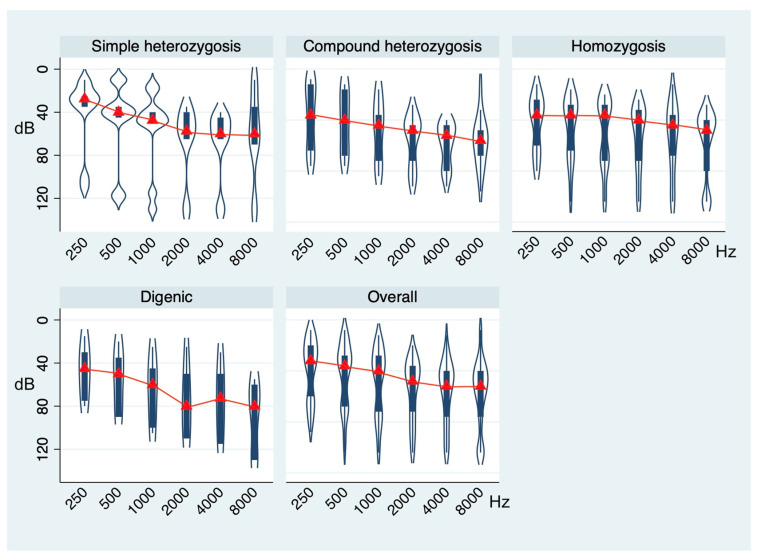
Violin plots showing the distribution of air-conduction thresholds at 250, 500, 1000, 2000, 4000, and 8000 Hz in the whole sample and across the genotype groups. *p*-value based on Roy’s largest-root test.

Comprehensively, no significant difference in terms of free-field and air-conduction PTAs was found across genotypes (Roy’s largest-root test, *p* = 0.9923 and *p* = 0.5785, respectively). However, considering the distribution of air-conduction thresholds at 250, 500, 1000, 2000, 4000, and 8000 Hz, the MANOVA model showed significantly different mean values across the different genotypes (Roy’s largest-root test, *p* = 0.0473). Figure 2 summarizes the hearing thresholds of cases stratified according to the four different genotypes.

In particular, on average, patients with simple heterozygosis showed moderate hearing loss (mean air-conduction PTA: 50.38 ± 27.59 dB) with a downsloping average audiometric configuration. Those in the compound heterozygosis group showed moderate-to-severe hearing loss (mean air-conduction PTA: 64.04 ± 25.57 dB) with a downsloping audiometric configuration. Patients with the homozygosis genotype showed severe hearing loss (mean air-conduction PTA: 63.70 ± 32.09 dB) with a flat average audiometric configuration, while those with the digenic mutation pattern had severe hearing loss (mean air-conduction PTA: 68.33 ± 31.93 dB) with a U-shaped average audiometric configuration.

**Figure 3:**
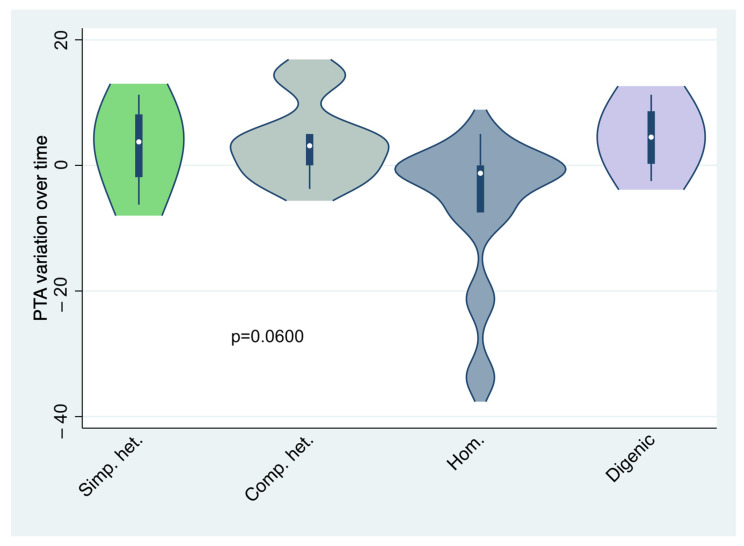
Violin plots showing the distribution of air-conduction PTA variation over time across the genotype groups. *p*-value is based on MANOVA model, with chi-squared approximation. Simp. het.: simple heterozygosis; Comp. het.: compound heterozygosis; Hom.: homozygosis.

No significant differences in terms of age at diagnosis, gender distribution, familiarity, and natural history of deafness (stable vs. progressive) could be found across different genotypes (Roy’s largest-root test: *p* = 0.8441; Fisher’s exact test: *p* = 0.849, *p* = 0.439, and *p* = 0.391, respectively; see also Table 1). In particular, regarding natural history of deafness, the mean air-conduction PTA change over time was not significantly different across the different genotypes (MANOVA model with chi-squared approximation, *p* = 0.0600; see also Table 1 and Figure 3).

## 4. Discussion

### 4.1. Genotype-to-Phenotype Association in GJB2 and GJB6 Genes Mutations

Hereditary hearing loss is clinically and genetically very heterogeneous. For many of the deafness genes, current knowledge on the associated phenotypes is far from complete. This is true in particular for those genes that have only recently been associated with HI. Phenotypic spectra are still also expanding for genes in which defects have long been known to underlie this condition [33].

Regarding the GJB2 and GJB6 genes mutations, the variability in auditory phenotypes seems to depend on the pleiotropic biological role of Connexin 26 and 30 in hearing transduction, and on the extent of the dysfunction of epithelial and connective-tissue gap junction networks in the cochlea [17]. Genotype-to-phenotype associations have long been investigated in an attempt to identify genotype-specific hearing loss patterns [23]. In 2005, Snoeckx et al. [23] investigated the phenotypes associated with different variants of GJB2 and GJB6. They found that truncating mutations of GJB2 were associated with a higher degree of HI than non-truncating mutations and that the pathogenicity of missense mutations depended on many factors, such as the position of the mutation within the protein and the nature of the aminoacidic substitution. In a recent review, Mao et al. [11] reported a complete and updated classification of the currently known associations between every missense GJB2 variant, the molecular mechanisms underlying hemichannel and gap junction functions, interactions with other co-expressed connexins like GJB6, and the clinical phenotypes. However, apart from highly prevalent mutations patterns, such as GJB2 (35delG/35delG), it might be difficult to obtain genetically homogeneous groups suitable to compare clinical data [23,34].

In accordance with the usual genotype distribution in Western countries, our series showed that GJB2 (35delG/35delG) was the most common mutation pattern, while the distribution of the other genotypes was scattered. For this reason, in this investigation, we considered the genotype pattern (simple heterozygosis, compound heterozygosis, homozygosis, and digenic mutation) rather than the individual mutation type. In our population, the five patients who had GJB2 mutations with simple heterozygosis showed the mildest degree of hearing impairment, with an average PTA of 50.38 ± 27.59 dB. This is consistent with the fact that, although the inheritance of most Connexin gene mutations has classically been considered to be autosomal-recessive, accumulating evidence has reported GJB2 mutations in heterozygosis to be a possible cause of congenital sensorineural hypoacusis [34]. In particular, patients presenting with GJB2 gene mutations in a heterozygosis pattern appear to show a milder phenotype in terms of hearing loss, seemingly due to the presence of a certain amount of wild-type Connexin 26 gene product, which may keep cochlear structures in a state of partially functioning [35,36]. In their review, Mao et al. [11] concluded that dominant-variant heterozygotes were mainly affected by milder post-lingual progressive HI, in accordance with the fact that the gap junction channel functions were only partially impaired. The compound heterozygosis group was quite heterogeneous in our series, comprising different mutations. Overall, the average PTA (64.04 ± 25.57 dB) showed severe hypoacusis with a sloping pattern. This was consistent with the most frequent overall hearing loss patterns across different GJB2 mutation types, as known from the literature [17,23,37]. According to this observation, Mao et al. [11] found that compound heterozygosity with missense variants that had serious consequences on the final functioning of gap junctions might lead to less severe hearing loss, while those that provided truncating variants could be associated with more severe audiological phenotypes. Regarding the homozygosis pattern, as previously reported, the majority of patients showed the GJB2 (35delG/35delG) mutation, the most frequent one in Western countries [17]. This probably reflected in the phenotype: in fact, in our sample, the homozygosis pattern was associated with an average PTA of 63.70 ± 32.09 dB, probably because of the prevalence of the GJB2 (35delG/35delG) mutation, which is known to be associated with severe-to-profound deafness [23]. It was observed [11] that non-syndromic HI due to GJB2 recessive missense variants was primarily congenital, bilateral, symmetric, non-progressive, and of a severe-to-profound degree. Regarding digenic mutations (GJB2/GJB6), as an associated phenotype, the literature reports severe-to-profound sensorineural hearing loss [23]. Snoeckx et al. [23] found the highest median PTA in a group of GJB2/GJB6 digenic heterozygotes, probably due to the fact that GJB6 partially substitutes GJB2 in inner ear function and leads to less efficient mechanisms with a more severe HI. This is substantially consistent with our data, which demonstrated the highest PTA values (68.33 ± 31.93 dB) in the digenic mutation group. However, although previous studies reported an average flat audiogram associated with digenic mutation patterns, in our series of patients, we found on average a U-shaped curve. This might be explained by the different distribution of digenic mutations (see also Table 1) in our cohort compared to previously described ones [23].

### 4.2. Open Issues in Congenital Hearing Loss Genetics

The etiological diagnosis of non-syndromic congenital hearing loss may provide information about the type of hearing loss and its evolution, allowing clinicians to inform patients and their families about heredity, natural history, and functional prognosis [36].

However, the diagnostic yield of genetic tests, even adopting a whole-exome sequencing approach, remains around 50% [38,39]. This means that, on average, half of all clinically identified cases remain without any genetic explanation. Moreover, cases in which the genotype seems not to be consistent with the phenotype exist, thus causing possible interpretation issues [40]. This is particularly relevant when the sequencing of the most frequently involved gene, namely GJB2, shows a mutation which is not known to cause hearing loss. In such cases, the analysis of other genes frequently mutated in individuals with non-syndromic hearing loss, such as STRC, MYO15A, MYO7A, TECTA, POU4F3, KCNQ4, SLC26A4, OTOF, MT-RNR1, MITF, and WFS1, or more extensive genetic testing through full-exome sequencing should be taken into consideration in order to both highlight possible synergies in determining the audiological phenotype and provide correct genetic counseling to parents [39,40]. Based on such approaches, many new insights, in addition to the extensive knowledge already obtained on the genetics of hearing loss, are expected.

### 4.3. Limitations and Strengths of This Study

The main limitations of the present study are as follows: (i) the monocentric nature, (ii) the retrospective design, and (iii) the limited patient numbers, especially in some subgroups, such as the simple heterozygosis one. On the other hand, the main strengths are mainly based on series homogeneity: (i) the audiological evaluation and diagnosis were performed by the same team with a standardized protocol; (ii) the genetic investigations were performed at the same laboratory; (iii) cases were homogeneously classified into subgroups according to the genotype patterns in terms of heterozygosis, compound heterozygosis, homozygosis, and digenic mutations; and (iv) the distribution of possible demographic and clinical confounding factors showed no significant difference across genotype groups.

## 5. Conclusions

In this study, the evaluation of hearing outcomes stratified by genotype led to the identification of different audiological patterns, which might reflect possible genotype-to-phenotype association in GJB2/GJB6 gene mutations. However, further prospective studies on large multi-centric series in different populations across the world are necessary to confirm these preliminary findings about the clinical and audiological features of patients with different mutation profiles.

## Figures and Tables

**Table 1:** Summary of the clinical features of the included cases across the genotype groups.

Variable	Total (N = 57)	Simple Heterozygosis (N = 5)	Compound Heterozygosis (N = 15)	Homozygosis (N = 34)	Digenic (N = 3)	*p*-Value
Age at diagnosis (months)	5.37	5.6	6.88	5.03	5.67	0.8441 *
Mean (SD)	(3.49)	(3.44)	(4.22)	(3.22)	(4.04)	
Gender						0.849 **
Male No. (%)	26 (45.61)	3 (60.00)	6 (40.00)	16 (47.06)	1 (33.33)	
Female No. (%)	31 (54.39)	2 (40.00)	9 (60.00)	18 (52.94)	2 (66.67)	
Familiarity						0.439 **
Familial No. (%)	19 (33.33)	2 (40.00)	7 (46.67)	10 (29.41)	0 (0.00)	
Non-familial No. (%)	38 (66.67)	3 (60.00)	8 (53.33)	24 (70.59)	3 (100.00)	
Place of birth						1.000 **
Italy No. (%)	45 (80.00)	4 (80.00)	12 (80.00)	26 (76.47)	3 (100.0)	
Abroad No. (%)	12 (20.00)	1 (20.00)	3 (20.00)	8 (23.53)	0 (0.00)	
Screening status						0.124 **
Pass No. (%)	5 (8.77)	1 (20.00)	1 (6.67)	3 (8.82)	0 (0.00)	
Fail No. (%)	21 (36.84)	0 (0.00)	3 (20.00)	17 (50.00)	1 (33.33)	
No data No. (%)	31 (54.39)	4 (80.00)	11 (73.33)	14 (41.18)	2 (66.67)	
Natural history						0.391 **
Progressive No. (%)	9 (15.79)	2 (40.00)	2 (13.33)	5 (14.71)	0 (0.00)	
Stable No. (%)	48 (84.21)	3 (60.00)	13 (86.67)	29 (85.29)	3 (100.00)	
PTA ^§^ open field	58.64	58.75	56.75	59.14	60.00	0.9923 *
Mean (SD)	(14.80)	(-)	(18.87)	(15.00)	(-)	
PTA ^§^ air conduction	61.98	50.38	64.04	63.70	68.33	0.5785 *
Mean (SD)	(29.28)	(27.59)	(25.57)	(32.09)	(31.93)	
Air–bone gap ^†^	5.92	4.83	8.12	5.09	4.38	0.7987 *
Mean (SD)	(6.57)	(0.80)	(11.63)	(0.91)	(1.25)	
Follow-up (months)	72.89	29.15	146.87	37.35	29.39	0.5147 *
Mean (SD)	(160.37)	(30.31)	(264.01)	(20.91)	(24.51)	
Mean PTA difference between diagnosis and last follow-up (dB)	0.01	3.13	3.88	−6.25	4.44	0.0600 ***
Mean (SD)	(9.66)	(7.25)	(6.28)	(11.42)	(5.75)	

No.: number; PTA: pure-tone average; SD: standard deviation; * MANOVA model based on Roy’s largest-root test; ** Fisher’s Exact test; ^§^ PTA based on 500, 1000, 2000, and 4000 Hz; ^†^ air–bone gap based on 500, 1000, 2000, and 4000 Hz; *** MANOVA model with chi-squared approximation.

## Data Availability

The datasets generated and analyzed during the current study are available on reasonable request. The data are not publicly available for privacy protection.
